# Inhibition of Lipoxygenases Showed No Benefit for the Musculoskeletal System in Estrogen Deficient Rats

**DOI:** 10.3389/fendo.2021.706504

**Published:** 2021-07-20

**Authors:** Dominik Saul, Friederike Eva Hohl, Max Konrad Franz, Ilka Meyer, Stefan Taudien, Paul Jonathan Roch, Stephan Sehmisch, Marina Komrakova

**Affiliations:** ^1^ Department of Trauma Surgery, Orthopaedics and Plastic Surgery, University Medical Center Goettingen, Goettingen, Germany; ^2^ Kogod Center on Aging and Division of Endocrinology, Mayo Clinic, Rochester, MN, United States; ^3^ Division of Infection Control and Infectious Diseases, Georg-August-University of Goettingen, Goettingen, Germany

**Keywords:** baicalein, bone healing, lipoxygenase-inhibitor, muscle tissue, osteoporosis, zileuton

## Abstract

**Background:**

In previous studies, we reported the beneficial impact of two lipoxygenase-inhibitors, Baicalein and Zileuton, on osteoporotic bone in a postmenopausal rat model. Whereas subcutaneous Baicalein predominantly improved cortical bone, Zileuton enhanced vertebral and femoral trabecular bone. In this study, we aimed to reveal whether the oral administration of Baicalein caused similar effects on bone and whether a combined administration of Baicalein and Zileuton could act synergistically to ameliorate the formerly reported effects in the musculoskeletal system.

**Methods:**

We treated ovariectomized (OVX) female Sprague-Dawley rats either with Baicalein (10mg/kg BW), Zileuton (10mg/kg BW) or a combination of both (each 10mg/kg BW) for 13 weeks and compared with untreated OVX and NON-OVX groups (n=12-16 rats per group). Lumbar vertebral bodies and femora were analyzed. Tibiae were osteotomized, plate-stabilized (at week 8 after OVX) and likewise analyzed by biomechanical, histological, micro-computed tomographical and ashing tests. The skeletal muscle structure was analyzed.

**Results:**

Oral administration of Baicalein did not confirm the reported favorable cortical effects in neither vertebra nor femur. Zileuton showed a beneficial effect on trabecular vertebra, while the femur was negatively affected. Callus formation was enhanced by all treatments; however, its density and biomechanical properties were unaltered. Lipoxygenase inhibition did not show a beneficial effect on skeletal muscle. The combination therapy did not ameliorate OVX-induced osteoporosis but induced even more bone loss.

**Conclusions:**

The preventive anti-osteoporotic treatments with two lipoxygenase inhibitors applied either alone or in combination showed no benefit for the musculoskeletal system in estrogen deficient rats.

## Introduction

With a prevalence of 200 million people worldwide, mostly postmenopausal women are affected by osteopenia or osteoporosis. In line with that, the annual amount of osteoporotic fractures among Medicare beneficiaries in the United States is estimated to be over 2.3 million (1–3). The “silent disease” loses covertness more and more due to extended guidelines and better prevention, but yet the long-term use of established medicamentous therapies causes several new dilemmas (4). Consequently and with a better understanding of underlying mechanisms that control bone turnover, new targets of therapy have been discovered and addressed (5). On this way, Receptor Activator of NF-κB Ligand (RANKL)- and Sclerostin-antibodies have found their way into the clinic (6, 7). However, advanced search for novel therapeutic approaches is ongoing.

Lipoxygenase (LOX) inhibitors have been found to suppress osteoclast formation and enhance bone formation *in vivo* (8, 9). A LOX inhibitor, Baicalein, which is extracted from the plant *Scutellaria baicalensis* inhibits 12- and 15-Lipoxygenase (*12*- and *15-LOX*). Another lipoxygenase inhibitor, Zileuton, inhibits *5-LOX* and has been FDA-approved (Zyflo^®^) for the treatment of asthma. Both lipoxygenase-inhibitors exert an antioxidative effect providing protection for cellular components and inhibiting apoptosis both in *in vitro* and *in vivo* studies (10–12).

In our previous studies we reported beneficial effects of these two LOX inhibitors applied as a mono-therapy for 4 to 5 weeks on bone parameters in the postmenopausal rat model. Baicalein was able to improve cortical bone, while Zileuton enhanced the trabecular structure in lumbar vertebrae and femora (13, 14). In addition to that, we demonstrated a beneficial effect of these treatments on the capillarization of the skeletal muscle (15). The bone healing process was also favorably affected by these LOX inhibitors (14, 16). Zileuton therapy improved cortical, callus and total bone volume at the osteotomy site in the tibia in ovariectomized rats (14), while a Baicalein monotherapy enhanced callus density without improving callus formation rate (16).

In the present study, we subsequently aimed to examine whether a combined therapy of Zileuton and Baicalein had an additional or interfering effect on cortical and trabecular bone in the lumbar spine and femur in an estrogen-deficient rat model. Furthermore, we elucidated the bone healing process in the tibia amongst these treatments and investigated their effects on skeletal muscle tissue. The LOX-inhibitors were applied orally to overcome the negative side effects of subcutaneous Baicalein administration (15) for a prolonged period of thirteen weeks, after the pharmacokinetics of Baicalein, orally administered in a concentration of 10 and 20 mg/kg, have been demonstrated to be reliably stable in rats by Kim et al. (17).

## Material and Methods

### Animals and Treatment

Seventy virgin female Sprague-Dawley rats (Janvier, Le Genest-Saint-Isle, France) were housed at 20°C and with a humidity of 55% in Makrolon^®^ IV cages (Techniplast Germany GmbH, Hohenpeissenberg, Germany). The experiments were conducted after approval of the local district government and in compliance with the ethical standards of animal care (no. 14/1530).

**Figure 1:**
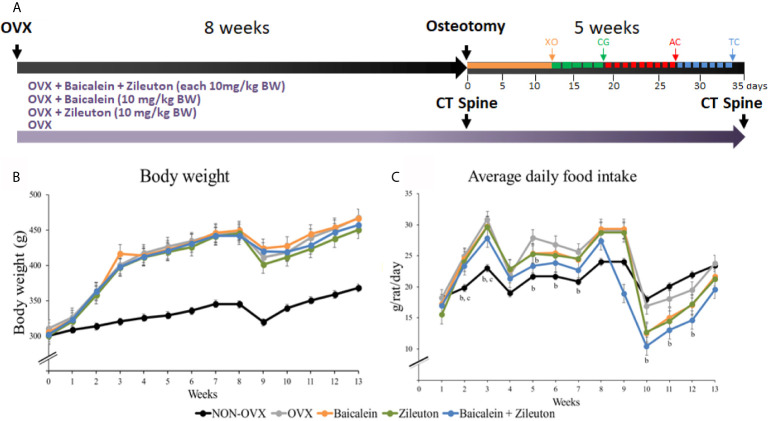
Graphical abstract of experimental setup. **(A)** After ovariectomy, 13 weeks-treatments with either Baicalein, Zileuton or their combination were conducted. Bilateral tibia osteotomy was performed 8 weeks after OVX. *In vivo* CT of the spine was executed after 8 and 13 weeks. On day 12, xylenol orange was administered, on day 19 calcein green, on day 27 alizarin complexone, followed by tetracycline on day 33. **(B)** The body weight of rats (g). **(C)** The average daily food intake (g/rat/day). ^b^Differs from OVX. ^c^Differs from Baicalein.

The rats were ovariectomized at the age of 3 months, one group (n=12) was left non-ovariectomized (NON-OVX). OVX rats were randomly divided into 4 groups and treated orally according to the group assignment for up to 13 weeks. One group was ovariectomized and left untreated (OVX, n=12), while another group was ovariectomized and received oral Baicalein therapy (10 mg/kg body weight [BW], Baicalein, n=15). The Zileuton-group was ovariectomized and received 10 mg/kg BW of Zileuton (Zileuton, n=15) and the other ovariectomized group received both Baicalein and Zileuton (each 10 mg/kg BW, Baicalein+Zileuton, n=16, **Figure 1A**).

A bilateral ovariectomy was performed as described earlier (13). Briefly, the surgical procedure was carried out under isoflurane anesthesia. Accordingly, anesthesia, shaving and disinfection were followed by incision on the abdomen bilaterally and dissection of adnexa before wound closure. Immediately after OVX, treatments with Baicalein and/or Zileuton were started. A soy-free diet [ssniff^®^ special diet; GmbH, Soest, Germany, ingredients listed in (13)] was supplemented either with Zileuton (Zyflo^®^; Cornerstone Therapeutics Inc., Cary, NC, USA) and/or Baicalein (98%; Sigma-Aldrich Chemie GmbH, Munich, Germany) at a concentration of 175 mg/kg food to achieve the dose of 10 mg/kg BW, respectively. This dose of Baicalein and Zileuton was shown to have an effect on bone tissue in previous studies (13–15, 18). During the experiment, all rats received soy-free diet either supplemented with Baicalein and/or Zileuton or without supplementation and demineralized water *ad libitum*.

Body weight and food intake were recorded on a weekly basis, with calculation of average daily food intake. The resulting effective dose averaged 10.35 ± 0.24 mg/kg BW in Baicalein group, 10.43 ± 0.22 mg/kg BW in Zileuton group and 10.48 ± 0.26 mg/kg BW (both) in Baicalein+Zileuton group. To ensure comparable amounts of food uptake, the individual rat weight was measured on a weekly basis (**Figures 1B, C**).

**Figure 2:**
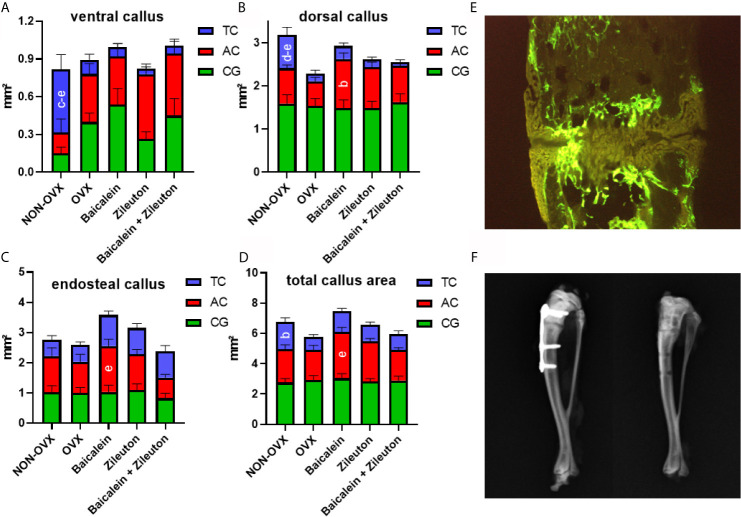
Tibia healing analysis. Ventral callus area **(A)**, dorsal callus area **(B)**, endosteal callus area **(C)** and total callus area **(D)** stained with fluorochromes XO+CG (0-19d after osteotomy), AC (20-27d after osteotomy) and TC (28-33d after osteotomy) and measured on histological sections **(E)**. X-rays prove the correct positioning of the plate after tibial osteotomy and healing is confirmed after plate removal **(F)**. [NON-OVX: n=8; OVX: n=10; Baicalein: n=9; Zileuton: n=11; Baicalein + Zileuton: n=11 (3 histological sections per animal)]. ^b^Differs from OVX. ^c^Differs from Baicalein. ^d^Differs from Zileuton. ^e^Differs from Baicalein+Zileuton (p < 0.05).

Eight weeks after OVX, a bilateral 0.5 mm osteotomy of the tibia metaphysis with consecutive 5-hole T-shaped titanium plate osteosynthesis (57-05140; Stryker Trauma, Selzach, Switzerland) was performed as described earlier (19) (**Figure 2F**). The osteotomy operation was performed under intraperitoneal anesthesia with 38 mg ketamine (Ketamin Inresa, Inresa Arzneimittel GmbH, Freiburg, Germany) and 2.5 mg midazolam (Rotexmedica GmbH, Trittau, Germany) per kg BW, respectively, along with isoflurane inhalation anesthesia (14, 19). Four rats died during osteotomy operation due to an anaesthesia side effect. After the osteotomy, rats were treated with 5 mg/kg BW Carprieve (Carprieve^®^ 50 mgl/ml Injekt, Norbrook Labs Ltd, Newry, Northern Ireland) twice a day for 5 days. Thereafter, 16 rats died due to the perforation of the intestine and the pain therapy was interrupted. No further losses were observed. Thus, precautions should be taken by applying analgesic treatments, since one of the side effects of NSAIDs is gastrointestinal damage (20). The administration of Carprieve once per day proved to be sufficient and had no severe side effects (19, 21, 22).

At the end of the experiment, the following numbers of animals were analyzed in NON-OVX group: 8, in OVX: 10, in Baicalein: 11, in Zileuton: 11 and in Baicalein+Zileuton 10.

Thirteen weeks after OVX, the rats were weighed, and decapitated after CO_2_-anesthesia. The uterus weight was recorded. Forth lumbar vertebral body (L4) was isolated, stored at -20°C for micro-computed tomography (micro-CT), ashing and compression test as described earlier (13). Right femora and right osteotomized tibiae were stored at -20^°^C for biomechanical, ashing and micro-CT analyses (23).

### *In Vivo* Quantitative Computed Tomography

Eight and thirteen weeks after OVX, *in vivo* pQCT of L4 was performed in isoflurane-anesthetized rats (n=5/group) using a pQCT device XCT Research SA (Stratec Medizintechnik GmbH, Pforzheim, Germany). The scans were performed in the same rats at both time points, with an exception in groups NON-OVX, OVX and Baicalein + Zileuton, where one rat in each group was replaced because of losses occurred after osteotomy. Bone mineral density (BMD, mg/cm³), total bone area (mm²) and stress-strain index (SSI) of L4 (central slice) and cross-sectional abdominal area (CSA, mm²) were measured as described in detail earlier (21). Three digital sections were analyzed for each animal. Analysis was performed using XCT-6.20C software (Stratec Medizintechnik GmbH, Pforzheim, Germany) as described earlier (13).

### Biomechanical Test

Biomechanical parameters of L4, right femora and right tibiae were assessed with a Zwick machine (145,660 Z020/TND; Ulm, Germany) (22–24). The L4 was fixed at the aluminum base and the stamp was loaded to the vertebral body (compression test). The femoral head was located in a 4 mm well on the end of the plate and loaded to the trochanteric region (breaking test). The tibia was placed on the aluminum base and loaded 2-3 mm distally to the osteotomy at tuberositas tibiae (bending test). A stamp mechanically dropped with 5 mm/min onto the bone with an initial force of 1 N. After this fixing procedure, measurements were performed with an accuracy of 0.2-0.4% within 2 and 500N and recorded with the aid of testXpert software (Zwick GmbH & Co. KG, Ulm, Germany) (13). The test was finalized after the initial linear curve increase declined more than 10N in L4 and femur and more than 2N in tibia. In femur, the test was performed until fracture occurred, whereas in tibia until plastic deformation of the bone. Reported values were the highest force, that the bone could withstand (Fmax, N) and slope of linear increment of the curve (Stiffness, N/mm), calculated using Microsoft Excel (Microsoft Corporation, Redmond, WA) (13).

### Micro-CT Analysis

To analyze cortical and trabecular parameters, L4, right femora and right tibiae were scanned using Quantum FX micro-CT (Caliper Sciences, Hopkinton, MA, USA). The scan protocol was as follows: 70 kVp, 200 µA, 30 Hz detector frame rate, 360° rotation, 3600 projections, 20x20 mm^2^ field of view, 512-pixel matrix and a 40 µm resolution. A phantom block with 5 defined hydroxyapatite elements (0.2, 0.4, 0.6, 0.8 and 1.0 g/cm³) accompanied every scan to convert grey values into bone mineral density (BMD) (16). 3D OsteoAnalyze software was used to calculate density and volume (16). Femoral region-of-interest was the head, which was cut in the transition zone from the *collum femoris* to the *trochanter major*. BV/TV, total BMD, cortical BMD (Ct.BMD), trabecular BMD (Tb.BMD), total and soft tissue volumes were measured (13, 25). Vertebral region-of-interest was the *corpus vertebra* as described earlier. Herein, we analyzed trabecular BMD (Tb.BMD), trabecular volume (Tb.V), total BMD and BV/TV (13). Tibial region-of-interest extended 1.5 mm proximally and distally from the osteotomy line (14). Analyzed values were cortical BMD (Ct.BMD), cortical volume (Ct.V), total bone BMD, total callus BMD (Cl.BMD) and osseous callus volume fraction (osseous Cl.V/total Cl.V) according to Bouxsein et al. (26). An exemplarily representation of the used thresholds with a NON-OVX rat for femur, tibia and lumbar vertebral body is shown in **Supplementary Figure 1**.

Further detailed structural analyses were performed on transformed 2D images. Three images of sagittal cut vertebral bodies, femora and tibiae were analyzed using MetaMorph Basic Acquisition Software (Leica Mikrosysteme Vertrieb GmbH, Wetzlar, Germany). Collected cortical data were thickness (Ct.Th, mm), area (Ct.Ar, mm^2^) and density (Ct. Dn, %). Trabecular parameters were density (Tb.Dn, %), number of nodes (N.Nd), density of trabecular nodes (N/mm²), trabecular thickness (Tb.Th, mm) and trabecular area (Tb.Ar, mm²). Callus parameters were density (Cl. Dn, %) and thickness (Cl. Th, mm) (13, 14, 16).

### Bone Healing Analysis

Dynamic callus formation was monitored using histological sections of right tibia stained in-vivo with fluorescent dyes: Xylenol orange (XO, 90 mg/kg BW), calcein green (CG, 10 mg/kg BW), alizarin complexone (AC, 30mg/kg BW) and tetracycline (TC, 25 mg/kg BW) were injected subcutaneously on days 12, 19, 27 and 33 after osteotomy, respectively (16) (**Figure 1A**). Briefly, tibia samples embedded in methyl methacrylate (Merck, Darmstadt, Germany) were cut longitudinally (150 µm thickness) using a diamond saw microtome (Leica SP1600, Leica Instruments GmbH, Nussloch, Germany). Subsequently, the 3 representative central sections were digitalized using a digital camera (Leica DC300F) and a zoom stereo microscope (Leica MZ75, Bensheim, Germany). The measurement area extended 2.5 mm from the osteotomy line and was divided into a ventral (plate side), dorsal (opposite side) and endosteal part. In each of these, the total callus area and labeling-specific callus areas were determined using the MetaMorph Basic Acquisition Software (Leica Mikrosysteme). XO and CG labeled callus areas were measured concurrently (XO+CG) (22).

### Ashing

For analysis of mineral content, L4 and right femur were ashed at 750°C for one hour in a muffle oven. Weight before ashing (organic) and after ashing (anorganic) were recorded (13, 27).

### Muscle Analysis

After sacrifice, Musculus gastrocnemius, soleus and longissimus were extracted and weighed. All muscles were frozen in liquid nitrogen and stored at -80°C for further histological analyses. Muscle from the right side were used for histological analyses. Samples were cut with a cryotome (12 µm sections,CM1900, Leica Microsystems). In Periodic acid-Schiff (PAS) stained sections, capillaries and fibers were counted manually within two 0.25 mm^2^ squares at 10x resolution. The ratio of capillaries to muscle fibers was analyzed (15). In ATPase stained sections, the diameter and area of single fibers in a 0.25mm² square were edged (n = 90 per fiber type) and determined using the NIS-Elements AR 4.0 program (Nikon Instruments Europe, Amsterdam, Netherlands). Type I and type IIb fibers were analyzed in combination (shown as type I fibers).

### Serum Analyses

After decapitation, serum samples were collected to detect levels of calcium, magnesium, phosphorus, alkaline phosphatase (Alp) activity, osteocalcin (OC) and RatLaps. The analyses were conducted at the Department of Clinical Chemistry, University of Goettingen using an Architect c16000 analyzer (Abbott, Wiesbaden, Germany) according to the manufacturer´s instructions.

OC was assessed with Rat-MID Osteocalcin EIA (Immunodiagnostic Systems [IDS], Frankfurt am Main, Germany). RatLaps was measured with RatLaps (CTX-I) EIA (IDS).

### Statistics

Statistical analysis was performed with GraphPad Prism 9.0 (GraphPad Software, San Diego, CA, USA) and R 4.0.2 (The R Foundation for Statistical Computing, Vienna, Austria). Normal distribution was assessed with Anderson-Darling test. One-way analysis of variance (ANOVA, p<0.05) was applied to detect the impact of the treatments. Differences between the groups were estimated by Tukey’s *post hoc* test with a significance level of 0.05 (95% confidence interval) (p<0.05). Data are presented as means and standard errors of the mean.

## Results

### LOX Inhibition Had No Effect on Body Weight, Uterus Weight, and Food Intake

While the body weight in the beginning was equally distributed among groups, all OVX groups had higher final weight compared to NON-OVX (**Table 1** and **Figures 1B, C**). Weight of uteri was significantly lower in all OVX groups compared to NON-OVX rats (**Table 1** and **Figures 1B, C**). The treatments with LOX inhibitors affected neither body weight nor uterus weight. The daily food intake was similarly distributed among the groups at the respective week (**Figure 1C**).

**Table 1:** Body weight, uterus weight, serum analyses, biomechanical and ashing analyses of bone.

	NON-OVX		OVX		Baicalein		Zileuton		Baicalein+Zileuton	
	Mean	SEM	Mean	SEM	Mean	SEM	Mean	SEM	Mean	SEM
** Weight**	n=8		n=10		n=11		n=11		n=10	
Body weight beginning (g)	300	4.0	310	4.5	306	3.3	300	4.6	302	4.2
Body weight end (g)	368^b-e^	7.5	467	11.8	467	7.0	451	7.9	458	15.3
Uterus weight (g)	0.57^b-e^	0.04	0.10	0.01	0.11	0.01	0.09	0.01	0.09	0.01
** Biomechanics - L4**	n=7		n=9		n=9		n=11		n=10	
Stiffness [N/mm]	351	28.2	298	22.5	282	18.2	339	16.6	327	19.7
Fmax [N]	326^b-e^	23.4	228	15.4	240	20.6	253	13.8	238	11.6
**Femur**	n=8		n=9		n=11		n=11		n=10	
Stiffness [N/mm]	387	30.3	334	19.1	324	25.8	343	24.5	299	19.9
Fmax [N]	194^b,e^	9.6	146	7.3	163	9.0	163	8.2	147	10.8
** Tibia**	n=7		n=10		n=11		n=11		n=9	
Stiffness [N/mm]	80	14.81	78	15.16	73	8.77	70	11.81	90	18.20
Fmax [N]	48	13.40	42	7.00	58	10.19	49	5.89	62	7.82
** Serum analysis**	n=8		n=10		n=11		n=11		n=10	
Calcium [mmol/l]	2.01	0.05	2.09	0.02	1.99	0.05	2.12	0.06	2.09	0.04
Magnesium [mmol/l	0.70	0.02	0.73	0.02	0.70	0.02	0.75	0.03	0.73	0.01
Phosphorus [mmol/l]	1.76	0.04	1.90	0.06	1.74	0.07	1.89	0.07	1.96	0.07
Alkaline phosphatase (Alp; U/l)	114	7.5	189^a^	15.1	154	11.8	176	22.7	157	13.7
Osteocalcin (OC; ng/ml)	116	5.0	147	11.5	177	18.0	170	19.3	211^a^	31.4
RatLaps (ng/ml)	5.47	1.22	9.48	0.91	9.69	1.64	9.70	1.84	10.67	1.77
** Ashing – L4**	n=8		n=10		n=11		n=11		n=10	
% organic content	67^c,d^	0.9	70	0.8	71	0.8	71	0.6	70	0.9
% anorganic content	33^c,d^	0.9	30	0.8	29	0.8	29	0.6	30	0.9
**Femur**	n=8		n=10		n=11		n=11		n=10	
% organic content	55^b-e^	0.7	59	0.8	58	0.8	59	0.7	61	1.3
% anorganic content	45^b-e^	0.7	41	0.8	42	0.8	41	0.7	39	1.3

^a^Differs from NON-OVX. ^b^Differs from OVX. ^c^Differs from Baicalein. ^d^Differs from Zileuton. ^e^Differs from Baicalein+Zileuton (p < 0.05).

### Combination Therapy Increased Serum OC Levels

The serum electrolytes calcium, magnesium and phosphorus did not differ significantly between the experimental groups (**Table 1**), whereas Alp was higher in OVX compared to NON-OVX group. RatLaps showed no differences, while OC was upregulated in combination therapy compared to NON-OVX control group, indicating a higher bone formation rate. The combination therapy increased serum OC levels but did not affect other serum parameters (**Table 1**).

### The Osteoporotic Phenotype Overweighs the Effect of LOX Inhibitors In In Vivo pQCT and Biomechanical Measurements

At both time points, before osteotomy (8 weeks after OVX) and at the end of the experiment, CSA was significantly higher in all OVX rats compared to NON-OVX irrespective of the treatments (**Table 2**). BMD of L4 was significantly lower in Baicalein- and Zileuton-treated groups after 8 weeks compared to NON-OVX (**Table 2**), whereas, at the end, all OVX groups showed lower BMD than NON-OVX rats (**Table 2**). The SSI was significantly higher in Zileuton compared to the OVX group (**Table 2**), while in other 3 OVX groups SSI was lower than in NON-OVX group at week 8 after OVX. At the end of the study, only the Baicalein group did not differ from NON-OVX, whereas SSI in the other OVX groups was significantly lower than in NON-OVX (**Table 2**).

**Table 2:** L4, *in vivo* pQCT.

	NON-OVX (n=5)		OVX (n=5)		Baicalein (n=5)		Zileuton (n=5)		Baicalein+Zileuton (n=5)	
	Mean	SEM	Mean	SEM	Mean	SEM	Mean	SEM	Mean	SEM
**CSA (mm²)**										
8 w after OVX	1886^b-e^	39.73	2306	39.91	2381	78.71	2380	32.38	2321	50.23
13 w after OVX	1866 ^b-e^	25.63	2284	58.66	2256	22.47	2438	41.57	2424	47.05
**BMD L4 (mg/cm³)**										
8 w after OVX	585.0^c,d^	6.9	551.2	10.3	531.5	9.1	536.1	12.4	547.5	9.1
13 w after OVX	554.8^b-e^	7.8	511.9	7.8	505.2	5.6	502.2	7.7	506.5	8.4
**SSI L4**										
8w after OVX	19.91^b,c,e^	0.75	15.59^d^	0.32	15.84	0.38	18.43	0.82	16.79	0.58
13w after OVX	18.21^b,d,e^	0.73	14.94	0.74	16.91	1.06	14.71	0.47	14.61	0.55

^b^Differs from OVX. ^c^Differs from Baicalein. ^d^Differs from Zileuton. ^e^Differs from Baicalein+Zileuton (p < 0.05).

The biomechanical analysis showed a reduction of Fmax in all OVX groups in vertebra and femur, but not of Fmax or stiffness in tibia, where no differences occurred (**Table 1**).

### Zileuton Ameliorated Trabecular Bone in L4, While Zileuton and Combined Therapies Deteriorated Trabecular Properties in the Femur

In L4, trabecular BMD, total BMD and BV/TV were reduced in all OVX groups irrespectively of the treatment (**Table 3**). In the femur, BV/TV was reduced in all OVX groups compared with the NON-OVX group (**Table 3**), whereas trabecular BMD was impaired in Baicalein and Baicalein+Zileuton groups and total BMD in OVX and Zileuton groups (**Table 3**). Summarizing, the micro-CT 3D analysis demonstrated a consistent effect of the ovariectomy in all treatment groups.

**Table 3:** micro-CT of L4, femora and tibiae, 3D and 2D analyses.

	NON-OVX		OVX		Baicalein		Zileuton		Baicalein+Zileuton	
	Mean	SEM	Mean	SEM	Mean	SEM	Mean	SEM	Mean	SEM
**L4, 3D**	n=8		n=10		n=10		n=11		n=10	
Tb. BMD (g/cm³)	0.634^b-e^	0.004	0.611	0.002	0.609	0.003	0.614	0.002	0.607	0.001
Total BMD (g/cm³)	0.606^b-e^	0.013	0.509	0.011	0.496	0.013	0.519	0.011	0.481	0.007
BV/TV (%)	70.67^b-e^	1.02	59.40	1.09	59.15	1.24	62.02	1.11	58.27	0.85
**L4,2D**	n=8		n=10		n=10		n=11		n=10	
Tb.Dn (%)	44.18^b-e^	1.126	26.37	0.6563	26.66	0.8595	31.44^b,c,e^	0.9328	27.86	0.7008
N.Nd (n)	40.63^b-e^	1.508	31.53	0.8033	29.70	1.387	34.79^b,c^	0.9342	32.52	0.9605
Tb.Th (mm)	0.25^b-e^	0.01	0.13^d^	0.01	0.14	0.01	0.17	0.01	0.15	0.01
Tb.Ar (mm²)	7.45^b-e^	0.23	4.84	0.11	4.80	0.17	5.85^b,c,e^	0.21	5.12	0.13
Ct.Dn (%)	94.43^d,e^	0.77	90.99	0.49	91.62	0.60	87.48	2.70	86.86	0.65
**Femur, 3D**	n=8		n=10		n=11		n=11		n=10	
Tb. BMD (g/cm³)	0.871^c,e^	0.006	0.854	0.003	0.848	0.007	0.852	0.004	0.849	0.004
Total BMD (g/cm³)	0.822^b,d^	0.021	0.663	0.017	0.726	0.029	0.695	0.026	0.690	0.014
BV/TV (%)	76.46^b-e^	1.68	60.65	2.46	65.27	2.49	65.53	2.38	64.09	1.22
**Femur, 2D**	n=8		n=10		n=11		n=11		n=10	
Tb.Dn (%)	49.18^b-e^	2.323	32.30^d,e^	1.995	29.96^e^	1.616	23.79	1.372	21.63	1.177
N.Nd (n)	73.54^b-e^	2.44	38.40^d,e^	2.71	32.87	2.32	30.18	1.55	20.52^c,d^	0.92
Tb.Th (µm)	121^b-e^	5.8	89	2.4	86	2.4	81	1.5	74^b,c^	1.4
Tb.Ar (mm²)	5.75^b-e^	0.18	3.67^c-e^	0.20	2.97	0.15	2.95	0.11	2.27^c,d^	0.09
Ct.Dn (%)	97.49	0.24	97.20	0.26	96.51	0.43	96.71	0.30	95.19^a,b,d^	0.52
**Tibia, 3D**	n=7		n=10		n=10		n=10		n=10	
Ct. BMD (g/cm³)	1.116^d,e^	0.034	1.083^d,e^	0.030	1.158	0.025	1.210	0.008	1.214	0.007
Ct.V (mm³)	20.40	5.158	14.43^d,e^	4.243	27.52	4.168	34.84	1.550	29.03	1.680
Total bone BMD (g/cm³)	0.585	0.025	0.519	0.024	0.522	0.018	0.532	0.021	0.496	0.019
Cl. BMD (g/cm³)	0.392^b-e^	0.012	0.326	0.017	0.333	0.005	0.331	0.007	0.327	0.012
Osseous Cl.V/Total Cl.V BV/TV	66.30^b,e^	2.19	57.67	2.534	59.18	1.09	59.16	1.50	55.98	1.77

^a^Differs from NON-OVX. ^b^Differs from OVX. ^c^Differs from Baicalein. ^d^Differs from Zileuton. ^e^Differs from Baicalein+Zileuton (p < 0.05).

Micro-CT 2D analysis revealed an improvement in L4 Tb.Dn, N.Nd and Tb.Th as well as Tb.Ar in Zileuton group compared to the OVX group in which an overall reduction of these parameters were observed compared to NON-OVX (**Table 3**). The cortical density was impaired in Zileuton and combination therapy in L4 (**Table 3**). In the femur, Zileuton and combination therapy led to reduced Tb.Dn and N.Nd compared to NON-OVX and OVX groups (**Table 3**), while the combination therapy significantly reduced Tb.Th, Tb.Ar and Ct.Dn (**Table 3**).

Taken together, sole treatment with Baicalein, did not change trabecular or cortical parameters compared to OVX (**Table 3**). The ovariectomy-induced negative effect on trabecular parameters could be rescued by Zileuton only therapy in L4, but in the femur the effect was contrary. The combination therapy had no effect on bone in L4, while in the femur, the diminishing effect of Zileuton remained after the addition of Baicalein.

### Organic Content Is Raised by Baicalein and Zileuton Therapies in L4

The organic content of L4 was significantly higher and, correspondingly, the anorganic content lower in Baicalein- and Zileuton only treated groups than in the NON-OVX group (**Table 1**). Organic content of femora was detected to be lower in the NON-OVX group than in all other groups, while the anorganic weight showed the opposite results (**Table 1**).

### Baicalein Accelerated Mid-Stage Callus Formation, While Zileuton and Combination Therapies Improved Cortical Parameters in the Tibia

Analyses of fluorochrome stained sections of the tibia (**Figure 2E**) revealed no differences in the time of the first osseous bridging of osteotomized bone ends (mean: 24 ± 1 day after osteotomy). The late ventral callus area (28-33d after osteotomy, TC staining) was significantly larger in NON-OVX animals compared to all other treatment groups (**Figure 2A**). Moreover, the late dorsal callus area was larger in NON-OVX compared to Zileuton and combination therapy groups (**Figure 2B**). Baicalein led to a larger midtime area (20-27d after osteotomy, AC staining) of dorsal callus area compared to the OVX group. Midtime endosteal callus area (AC, **Figure 2C**) and total callus area (**Figure 2D**) were larger in the Baicalein group than in the combination therapy group. The late total callus area (TC staining) in the NON-OVX group exceeded the OVX group (**Figure 2D**).

Micro-CT 3D analysis showed enhanced Ct. BMD and Ct.V in the tibia in both Zileuton and combination therapy groups (**Table 3**), while total Cl. BMD was reduced in all OVX groups (**Table 3**). The osseous callus volume fraction, however, was lowest in the combination therapy group (**Table 3**), while total bone BMD did not differ among the groups (**Table 3**).

Summarizing, Baicalein treatment resulted in enhanced endosteal and total callus area compared to combination therapy and increased dorsal callus area compared to the OVX group. The cortical parameters in the tibia were enhanced by Zileuton and combination therapy compared to OVX.

### Combination Therapy Enhanced Capillary Ratio in M. Gastrocnemius

In Musculus gastrocnemius, soleus and longissimus neither diameter nor area of muscle fibers differed significantly among treatment groups (**Table 4**). The combination therapy in M. gastrocnemius led to a higher capillary ratio compared to NON-OVX and OVX groups, which was not noticeable in M. longissimus and M. soleus (**Table 4**).

**Table 4:** Histological analyses of M. Gastrocnemius (MG), M. Longissimus (ML) and M. Soleus (MS).

	NON-OVX (n=8)		OVX (n=10)		Baicalein (n=10)		Zileuton (n=11)		Baicalein+Zileuton (n=10)	
	Mean	SEM	Mean	SEM	Mean	SEM	Mean	SEM	Mean	SEM
**MG, type I**										
Diameter (µm)	48.91	2.76	50.91	1.79	51.42	1.22	50.25	1.75	50.01	1.72
Area (µm²)	1939	222	2094	135	2094	101	2048	148	2034	135
**MG, type IIa**										
Diameter (µm)	71.84	2.54	76.42	2.25	77.17	2.38	73.29	1.28	72.78	2.44
Area (µm²)	4153	309	4699	277	4790	289	4285	154	4098	319
**MG, capillarization**										
Capillary/Fiber	1.22	0.05	1.27	0.04	1.45	0.075	1.39	0.077	1.67^a,b^	0.10
**ML, type I**										
Diameter (µm)	56.64	1.83	56.84	1.16	54.46	1.94	57.16	1.25	54.55	2.21
Area (µm²)	2619	168	2606	109	2390	176	2610	117	2372	183
**ML, type IIa**										
Diameter (µm)	82.81	0.84	88.71	1.86	81.90	3.18	84.99	1.62	83.32	3.09
Area (µm²)	5442	115	6248	317	5387	412	5725	230	5551	407
**ML, capillarization**										
Capillary/Fiber	1.15	0.09	1.38	0.07	1.46	0.08	1.42	0.07	1.38	0.08
**MS, type I**										
Diameter (µm)	68.39	1.33	66.93	2.48	67.50	1.34	67.73	1.50	66.90	0.73
Area (µm²)	3733	145.9	3604	287.5	3628	146.9	3665	168.5	3554	76.18
**MS, capillarization**										
Capillary/Fiber	1.57	0.06	1.81	0.08	1.57	0.06	1.59	0.07	1.71	0.11

^a^Differs from NON-OVX. ^b^Differs from OVX.

## Discussion

Osteoporosis as a systemic disease is manifested on both cortical and trabecular bone. The detection of osteoporosis as well as the therapeutic success are closely connected to the structural changes in spine and proximal femur (28, 29). Due to an aging society, an increasing socioeconomic relevance is attributed to this “silent disease” and the need for new therapeutic approaches is evident (5, 30, 31). Recently, we have reported promising results of sole Baicalein and Zileuton treatments on osteoporotic bone tissue (13, 14, 16), though some undesirable side-effects resulted from subcutaneous administration of the former. We subsequently decided on an oral administration of both LOX inhibitors. A oral administration of Baicalein in a dose of 10mg/kg per day exhibited inhibitory effects in a xenograft experiment in mice (32), while in a rat experiment, an oral dose of 10 and 20mg/kg Baicalein led to a stable concentration of its active metabolite, Baicalin, in the rat plasma (17).

In the present study, we investigated the effect of a combined treatment of Baicalein and Zileuton as well as single treatments of these substances on lumbar vertebral body and femur, and on bone healing in the osteotomized tibia as well as on the skeletal muscle in an ovariectomized rat model of postmenopausal osteoporosis (13, 22). Effects of the LOX inhibitors on body weight, uterus weight or food intake were ruled out since the treatments did not lead to differences in these compared to the OVX group. We did not detect effects of these substances on the whole body composition in our previous studies either (13, 16, 19).

Previously, we detected favorable effects on the capillarization of muscle after subcutaneous injection of Baicalein (15) and oral administration of Zileuton (unpublished data) for up to 4-5 weeks. In the present study, the effect was less pronounced. Solely the combination therapy increased capillarization in M. gastrocnemius. One reason might be that the muscular effect is short-lasting, leading to detectable effects after short-term treatments [as in our previous study (15)], but diminishing due to adaption after 13 weeks. Another possible reason might root in the time of administration. In this study, the LOX inhibitors were applied as preventive treatments, immediately after OVX, whereas previously they were applied 8 weeks after OVX when changes in the musculoskeletal system due to hormone deficiency had already manifested (33).

In our *in vivo* pQCT, no effect of LOX inhibitors was observed on CSA. The combination therapy did not change SSI of L4, whereas Zileuton-treatment showed higher SSI than that in the OVX group after an 8 week treatment, which is comparable to our previous 5 week treatment (13). The oral route of administration for Baicalein was demonstrated to be safe, however it showed no effect on BMD and SSI in the *in vivo* pQCT analyses. The micro-CT 3D analysis revealed no effect of either therapy on bone parameters, whereas the 2D analysis illustrated a beneficial effect of Zileuton on trabecular bone parameters in the lumbar spine. Thus, the previously reported trabecular accentuated effects of Zileuton on the vertebra (13) could again be confirmed after the longer treatment regimen for 13 weeks in this study. In contrast to these findings in L4, the trabecular bone was impaired in femur after Zileuton and combination treatments. Furthermore, the cortical bone lost its density after these treatments in L4, too. After a short-term application, the Zileuton treatment caused an increase of bone volume in the femur, whereas femoral density was reduced (14). An oral Baicalein treatment did not change parameters measured by micro-CT in the present study. Hence, we supported our previous findings of Zileuton being favorable in trabecular bone in spine and unfavorable in femur (13), while Baicalein did not show any effect on bone and did not reduce the negative effect of Zileuton in the combination therapy in femur.

Proximal femur fractures, which occur commonly in the aged people, constitute a devastating diagnosis with 1-year mortality rates of up to 36%, regularly indicating missed anti-osteoporotic therapy (34). The previously reported effects of Baicalein were sparse (16), while cortical volume in the distal femur was significantly ameliorated after Zileuton-therapy in a dose of 10mg/kg BW (14). Three-dimensional analysis of femoral head confirmed weak effects of Baicalein. The advantage of Zileuton on cortical volume was not consistent after prolonged therapy in our study. The combination therapy, however, reduced Tb.Th and Ct.Dn substantially. All of these findings put the combination therapy in an unfavorable light.

In our bone healing analysis, a favorable effect of sole Baicalein therapy was detected in the dorsal callus area, mostly in the midterm healing period. Indeed, the main effect of both Baicalein and Zileuton as their combination therapy on ventral and dorsal callus formation was observed within 20 and 33 days after osteotomy. In a former study, we could detect an impairment of early callus formation after subcutaneous Baicalein administration (16). The oral treatment with either Baicalein, Zileuton or both in the current study showed a reduction in these groups in the late ventral callus formation, whereas the cortical parameters were improved after Zileuton und combination therapies at the osteotomy site. Contrary to our descriptions of a partial deterioration of fracture healing *via* pharmacological lipoxygenase-inhibition, the positive effect of a lipoxygenase knockout on fracture healing was demonstrated in a *5-LO*(KO) mouse model by Manigrasso et al. (29). In their knockout model, the authors observed enhanced healing with substantially better mechanical properties of the newly formed bone compared to wild type mice (35).

However, the exact underlying molecular mechanism of Baicalein’s mode of action is not understood in detail. Several key enzymes like *5*-, *12*- and *15-LOX* are inhibited next to cyclooxygenases (*COX 1* and *2*), resulting in reduced synthesis of prostaglandins (36, 37). Intriguingly, both Zileuton and Baicalein have been demonstrated to inhibit stress-mediated 5-LOX metabolite (cysteinyl leukotrienes) production, which may be a reason for the lack of synergistic effects in our study (36). Besides, Baicalein was demonstrated to inhibit parathormone (PTH)-induced DNA synthesis and cytokines like *IL-6* and *TNF-α* (38, 39). Further stimulating osteoblast differentiation, the induction of *NF-κB* and *nuclear factor of activated T-cells, cytoplasmic 1* (*NFATc1*) as well as the induction of osteoclast apoptosis have been described, resulting in an overall osteogenic effect (40, 41). In our previous study, we speculated that Baicalein had an antioxidative effect next to osteoclast apoptosis (via Wnt/β-catenin pathway) and radical-scavenging effect (16). The main effect of Zileuton is created *via 5-LOX* inhibition. While *in vivo* data show modulation of cytokine release and reduced osteoclast differentiation (42), additionally giving rise to osteoanabolic reactions, our own analysis showed an upregulation of *osteocalcin* and *Alp* in the serum (14, 42). Despite the bone healing analyses showed no sweeping effects of Zileuton and combination therapy on callus density, callus area and cortical bone (Ct.BMD and Ct.V), the healed cortical tibia (Ct.BMD, Ct.V) was favorably affected by these treatments. A connecting role may be attributed to the raised serum levels of *osteocalcin* which we measured in combination therapy. These have been similarly described after 5-LOX inhibition elsewhere (16, 43).

## Conclusion

Summarizing, the spinal trabecular bone was improved by Zileuton, but not by combination therapy, which is even in disfavor for Tb.BMD, BV/TV and Ct.Dn. The analysis of the clinically crucial proximal femur could not show the expected ameliorative influence of LOX inhibitors, but instead clarified plain negative consequences of combined therapy of Baicalein and Zileuton on cortical and trabecular bone. The oral administration of Baicalein - despite a longer therapy period – did not lead to the same beneficial cortical effects as described earlier (16). Though the dynamic bone healing process was favorably affected by all LOX inhibitor treatments, their overall effect on bone healing was minor. Furthermore, inhibition of LOX did not have a profound benefit on the skeletal muscle.

Taken together, our data showed no perspective of both lipoxygenase inhibitors neither alone or in combination for the musculoskeletal system in estrogen deficient rats and particularly not as a preventive treatment of osteoporosis.

### Limitations

The oral administration of Baicalein instead of the previously reported subcutaneous administration - due to reported negative side effects - might have affected the results (15). The lack of assessment of metabolic parameters in our rat model yet limits the mechanistic insights of Baicalein and Zileuton action.

## Data Availability Statement

The raw data supporting the conclusions of this article will be made available by the authors, without undue reservation.

## Ethics Statement

The animal study was reviewed and approved by the the local district government (Oldenburg, Germany) and in compliance with the ethical standards of animal care.

## Author Contributions

MK and SS designed the study. FH, MF, IM, and ST performed all experimental procedures. Data analysis was carried out by DS and MK. DS wrote the manuscript with the help of MK. DS, FH, MF, IM, ST, PR, SS, and MK critically revised it for important intellectual content. All authors contributed to the article and approved the submitted version.

## Funding

DS was funded by the Deutsche Forschungsgemeinschaft (DFG, German Research Foundation) – 413501650. We thank the Elsbeth Bonhoff Stiftung for financial support (Grant N114).

## Conflict of Interest

The authors declare that the research was conducted in the absence of any commercial or financial relationships that could be construed as a potential conflict of interest.

## References

[1] Reginster J-Y Burlet N. Osteoporosis: A Still Increasing Prevalence. Bone (2006) 38:S4–9. 10.1016/j.bone.2005.11.024 16455317

[2] Watts NB Bilezikian JP Camacho PM Greenspan SL Harris ST Hodgson SF. American Association of Clinical Endocrinologists Medical Guidelines for Clinical Practice for the Diagnosis and Treatment of Postmenopausal Osteoporosis. Endocr Pract (2010) 16(Suppl 3):1–37. 10.4158/EP.16.S3.1 PMC4876714 21224201

[3] Hansen D Bazell C Pelizzari P Pyenson B. Medicare Cost of Osteoporotic Fractures: The Clinical and Cost Burden of an Important Consequence of Osteoporosis (2020). Available at: https://static1.squarespace.com/static/5c0860aff793924efe2230f3/t/5d76b949deb7e9086ee3d7dd/1568061771769/Medicare+Cost+of+Osteoporotic+Fractures+20190827.pdf. (Accessed March 08, 2021).

[4] Brown C. Osteoporosis: Staying Strong. Nature (2017) 550:S15–7. 10.1038/550S15a 28976955

[5] Makras P Delaroudis S Anastasilakis AD. Novel Therapies for Osteoporosis. Metabolism (2015) 64:1199–214. 10.1016/j.metabol.2015.07.011 26277199

[6] Cosman F Crittenden DB Adachi JD Binkley N Czerwinski E Ferrari S. Romosozumab Treatment in Postmenopausal Women With Osteoporosis. N Engl J Med (2016) 375:1532–43. 10.1056/NEJMoa1607948 27641143

[7] Cummings SR San Martin J McClung MR Siris ES Eastell R Reid IR. Denosumab for Prevention of Fractures in Postmenopausal Women With Osteoporosis. N Engl J Med (2009) 361:756–65. 10.1056/NEJMoa0809493 19671655

[8] Kang J-H Ting Z Moon M Sim J-S Lee J-M Doh K-E. 5-Lipoxygenase Inhibitors Suppress RANKL-Induced Osteoclast Formation *Via* NFATc1 Expression. Bioorg Med Chem (2015) 23:7069–78. 10.1016/j.bmc.2015.09.025 26432605

[9] Cottrell JA Keshav V Mitchell A O’Connor JP. Local Inhibition of 5-Lipoxygenase Enhances Bone Formation in a Rat Model. Bone Joint Res (2013) 2:41–50. 10.1302/2046-3758.22.2000066 23610701 PMC3626215

[10] Kang KA Zhang R Piao MJ Chae S Kim HS Park JH. Baicalein Inhibits Oxidative Stress-Induced Cellular Damage *Via* Antioxidant Effects. Toxicol Ind Health (2012) 28:412–21. 10.1177/0748233711413799 21957089

[11] Wu Q-Q Deng W Xiao Y Chen J-J Liu C Wang J. The 5-Lipoxygenase Inhibitor Zileuton Protects Pressure Overload-Induced Cardiac Remodeling *Via* Activating PPARα. Oxid Med Cell Longev (2019) 2019:7536803. 10.1155/2019/7536803 31781348 PMC6874937

[12] Shi L Hao Z Zhang S Wei M Lu B Wang Z. Baicalein and Baicalin Alleviate Acetaminophen-Induced Liver Injury by Activating Nrf2 Antioxidative Pathway: The Involvement of ERK1/2 and PKC. Biochem Pharmacol (2018) 150:9–23. 10.1016/j.bcp.2018.01.026 29338970

[13] Saul D Gleitz S Nguyen HH Kosinsky RL Sehmisch S Hoffmann DB. Effect of the Lipoxygenase-Inhibitors Baicalein and Zileuton on the Vertebra in Ovariectomized Rats. Bone (2017) 101:134–44. 10.1016/j.bone.2017.04.011 28455215

[14] Saul D Ninkovic M Komrakova M Wolff L Simka P Gasimov T. Effect of Zileuton on Osteoporotic Bone and its Healing, Expression of Bone, and Brain Genes in Rats. J Appl Physiol (Bethesda Md 1985) (2018) 124:118–30. 10.1152/japplphysiol.01126.2016 28860177

[15] Saul D Kling JH Kosinsky RL Hoffmann DB Komrakova M Wicke M. Effect of the Lipoxygenase Inhibitor Baicalein on Muscles in Ovariectomized Rats. J Nutr Metab (2016) 2016:3703216. 10.1155/2016/3703216 28050282 PMC5165164

[16] Saul D Weber M Zimmermann MH Kosinsky RL Hoffmann DB Menger B. Effect of the Lipoxygenase Inhibitor Baicalein on Bone Tissue and Bone Healing in Ovariectomized Rats. Nutr Metab (2019) 16:4. 10.1186/s12986-018-0327-2 PMC6329162 30651746

[17] Kim YH Jeong DW Kim Y-C Sohn DH Park E-S Lee HS. Pharmacokinetics of Baicalein, Baicalin and Wogonin After Oral Administration of a Standardized Extract of Scutellaria Baicalensis, PF-2405 in Rats. Arch Pharm Res (2007) 30:260–5. 10.1007/BF02977703 17366750

[18] Kim DH Hossain MA Kang YJ Jang JY Lee YJ Im E. Baicalein, an Active Component of Scutellaria Baicalensis Georgi, Induces Apoptosis in Human Colon Cancer Cells and Prevents AOM/DSS-induced Colon Cancer in Mice. Int J Oncol (2013) 43:1652–8. 10.3892/ijo.2013.2086 24008356

[19] Komrakova M Fiebig J Hoffmann DB Krischek C Lehmann W Stuermer KM. The Advantages of Bilateral Osteotomy Over Unilateral Osteotomy for Osteoporotic Bone Healing. Calcif Tissue Int (2018) 103:80–94. 10.1007/s00223-018-0392-6 29352329

[20] Smolinske SC Hall AH Vandenberg SA Spoerke DG McBride PV. Toxic Effects of Nonsteroidal Anti-Inflammatory Drugs in Overdose. An Overview of Recent Evidence on Clinical Effects and Dose-Response Relationships. Drug Saf (1990) 5:252–74. 10.2165/00002018-199005040-00003 2198051

[21] Komrakova M Hoffmann DB Nuehnen V Stueber H Wassmann M Wicke M. The Effect of Vibration Treatments Combined With Teriparatide or Strontium Ranelate on Bone Healing and Muscle in Ovariectomized Rats. Calcif Tissue Int (2016) 99:408–22. 10.1007/s00223-016-0156-0 27272029

[22] Komrakova M Weidemann A Dullin C Ebert J Tezval M Stuermer KM. The Impact of Strontium Ranelate on Metaphyseal Bone Healing in Ovariectomized Rats. Calcif Tissue Int (2015) 97:391–401. 10.1007/s00223-015-0019-0 26084691

[23] Tezval M Stuermer EK Sehmisch S Rack T Stary A Stebener M. Improvement of Trochanteric Bone Quality in an Osteoporosis Model After Short-Term Treatment With Parathyroid Hormone: A New Mechanical Test for Trochanteric Region of Rat Femur. Osteoporos Int (2010) 21:251–61. 10.1007/s00198-009-0941-y PMC2801842 19436940

[24] Sehmisch S Erren M Rack T Tezval M Seidlova-Wuttke D Richter J. Short-Term Effects of Parathyroid Hormone on Rat Lumbar Vertebrae. Spine (2009) 34:2014–21. 10.1097/BRS.0b013e3181afe846 19730209

[25] Parfitt AM Drezner MK Glorieux FH Kanis JA Malluche H Meunier PJ. Bone Histomorphometry: Standardization of Nomenclature, Symbols, and Units. Report of the ASBMR Histomorphometry Nomenclature Committee. J Bone Miner Res (1987) 2:595–610. 10.1002/jbmr.5650020617 3455637

[26] Bouxsein ML Boyd SK Christiansen BA Guldberg RE Jepsen KJ Müller R. Guidelines for Assessment of Bone Microstructure in Rodents Using Micro-Computed Tomography. J Bone Miner Res (2010) 25:1468–86. 10.1002/jbmr.141 20533309

[27] Komrakova M Stuermer EK Sturm A Schmelz U Tezval M Stuermer KM. Efficiency of 48h vs. 24h Injection of Parathyroid Hormone for Amelioration of Osteopenic Spine Properties in Male Rats. Open Bone J (2012) 4:20–6. 10.2174/1876525401204010020

[28] Siris ES Adler R Bilezikian J Bolognese M Dawson-Hughes B Favus MJ. The Clinical Diagnosis of Osteoporosis: A Position Statement From the National Bone Health Alliance Working Group. Osteoporos Int (2014) 25:1439–43. 10.1007/s00198-014-2655-z PMC3988515 24577348

[29] Wright NC Saag KG Dawson-Hughes B Khosla S Siris ES. The Impact of the New National Bone Health Alliance (NBHA) Diagnostic Criteria on the Prevalence of Osteoporosis in the USA. Osteoporos Int (2017) 28:1225–32. 10.1007/s00198-016-3865-3 27966104

[30] Rachner TD Khosla S Hofbauer LC. Osteoporosis: Now and the Future. Lancet (London England) (2011) 377:1276–87. 10.1016/S0140-6736(10)62349-5 PMC3555696 21450337

[31] Watts NB Bilezikian JP. Advances in Target-Specific Therapy for Osteoporosis. J Clin Endocrinol Metab (2014) 99:1149–51. 10.1210/jc.2014-1065 24446660

[32] Miocinovic R McCabe N Keck RW Jankun J Hampton JA Selman SH. *In Vivo* and *In Vitro* Effect of Baicalein on Human Prostate Cancer Cells. Int J Oncol (2005) 26(1):241–6. 10.3892/ijo.26.1.241 15586246

[33] Yousefzadeh N Kashfi K Jeddi S Ghasemi A. Ovariectomized Rat Model of Osteoporosis: A Practical Guide. EXCLI J (2020) 19:89–107. 10.17179/excli2019-1990 32038119 PMC7003643

[34] Bhandari M Swiontkowski M. Management of Acute Hip Fracture. N Engl J Med (2017) 377:2053–62. 10.1056/NEJMcp1611090 29166235

[35] Manigrasso MB O’Connor JP. Accelerated Fracture Healing in Mice Lacking the 5-Lipoxygenase Gene. Acta Orthop (2010) 81:748–55. 10.3109/17453674.2010.533931 PMC3216088 21067431

[36] Li C Zhang W Fang S Lu Y Zhang L Qi L. Baicalin Attenuates Oxygen-Glucose Deprivation-Induced Injury by Inhibiting Oxidative Stress-Mediated 5-Lipoxygenase Activation in PC12 Cells. Acta Pharmacol Sin (2010) 31:137–44. 10.1038/aps.2009.196 PMC4002841 20139896

[37] Chen S. Natural Products Triggering Biological Targets–a Review of the Anti-Inflammatory Phytochemicals Targeting the Arachidonic Acid Pathway in Allergy Asthma and Rheumatoid Arthritis. Curr Drug Targets (2011) 12:288–301. 10.2174/138945011794815347 20955151

[38] Somjen D Tordjman K Katzburg S Knoll E Sharon O Limor R. Lipoxygenase Metabolites are Mediators of PTH-Dependent Human Osteoblast Growth. Bone (2008) 42:491–7. 10.1016/j.bone.2007.11.005 18187376

[39] Hu S Chen Y Wang Z-F Mao-Ying Q-L Mi W-L Jiang J-W. The Analgesic and Antineuroinflammatory Effect of Baicalein in Cancer-Induced Bone Pain. Evid Based Complement Altern Med (2015) 2015:973524. 10.1155/2015/973524 PMC4662985 26649065

[40] Kim JM Lee S-U Kim YS Min YK Kim SH. Baicalein Stimulates Osteoblast Differentiation *Via* Coordinating Activation of MAP Kinases and Transcription Factors. J Cell Biochem (2008) 104:1906–17. 10.1002/jcb.21760 18384125

[41] Kim MH Ryu SY Bae MA Choi J-S Min YK Kim SH. Baicalein Inhibits Osteoclast Differentiation and Induces Mature Osteoclast Apoptosis. Food Chem Toxicol (2008) 46:3375–82. 10.1016/j.fct.2008.08.016 18786594

[42] Moura AP Taddei SR Queiroz-Junior CM Madeira MF Rodrigues LF Garlet GP. The Relevance of Leukotrienes for Bone Resorption Induced by Mechanical Loading. Bone (2014) 69:133–8. 10.1016/j.bone.2014.09.019 25270168

[43] Cottrell JA O’Connor JP. Pharmacological Inhibition of 5-Lipoxygenase Accelerates and Enhances Fracture-Healing. J Bone Joint Surg Am (2009) 91:2653–65. 10.2106/JBJS.H.01844 19884440

